# Effects of Mandibular Retrusive Deviation on Prefrontal Cortex Activation: A Functional Near-Infrared Spectroscopy Study

**DOI:** 10.1155/2015/373769

**Published:** 2015-05-17

**Authors:** Takero Otsuka, Ryuichi Yamasaki, Tateshi Shimazaki, Fumihiko Yoshino, Kenichi Sasaguri, Toshitsugu Kawata

**Affiliations:** ^1^Orthodontic Division, Department of Oral Science, Kanagawa Dental University Graduate School, 82 Inaoka-cho, Yokosuka, Kanagawa 238-8580, Japan; ^2^Photomedical Dentistry Division, Department of Oral Science, Kanagawa Dental University Graduate School, 82 Inaoka-cho, Yokosuka, Kanagawa 238-8580, Japan

## Abstract

The objective of this study was to evaluate occlusal condition by assessing brain activity in the prefrontal cortex, which is associated with emotion. Functional near-infrared spectroscopy (fNIRS) was used to detect changes in cerebral blood flow in the prefrontal cortex of 12 healthy volunteers. The malocclusion model was a custom-made splint that forced the mandible into retrusion. A splint with no modification was used as a control. The cortical activation during clenching was compared between the retrusive position condition and the control condition. A visual analog scale score for discomfort was also obtained during clenching and used to evaluate the interaction between fNIRS data and psychiatric changes. Activation of the prefrontal cortex was significantly greater during clenching in the mandibular retrusive condition than during clenching in the control condition. Furthermore, Spearman rank-correlation coefficient revealed a parallel relation between prefrontal cortex activation and visual analog scale score for discomfort. These results indicate that fNIRS can be used to objectively evaluate the occlusal condition by evaluating activity in the prefrontal cortex.

## 1. Introduction

Some studies have reported that occlusal dysfunction affects the stress response via changes in brain activity and leads to nonoral health problems [1–6].We have often seen the mandibular retrusive condition that is one form of malocclusal condition in dental practice. And that malocclusal condition is often caused by inappropriate dental treatment. Our previous functional magnetic resonance imaging (fMRI) studies have suggested that mandibular retrusion affects the emotional state of brain regions [1, 2]. In addition, prefrontal cortex (PFC) activity correlates with the severity of malocclusion. In this respect, measuring brain activity could provide an objective measure of occlusal dysfunction in humans.

Current methods used to measure brain activity in medical and clinical research include positron emission tomography, magnetoencephalography, fMRI, and functional near-infrared spectroscopy (fNIRS). These neuroimaging techniques have also been used in the field of dentistry to study the effects of various oral conditionson the brain [1, 2, 7, 8]. However, the results of these researches have yet to be translated into clinical dental applications, as the above-mentioned systems are often expensive and difficult to operate and fMRI and magnetoencephalography require the patient's head to be secured and the patient's posture to be limited. However, fNIRS is a noninvasive technique that measures brain activity via changes in cerebral blood flow (mainly in the cerebral cortex) by monitoring hemoglobin concentration using near-infrared light [9]. fNIRS devices can be miniaturized to suit specific purposes, allowing brain activity to be assessed with the subject in any body position and without the subject having to remain perfectly still. Therefore, fNIRS is more suitable for clinical application than other neuroimaging methods. Several fNIRS studies have suggested that fNIRS of the prefrontal cortex (PFC) can evaluate the human emotional state for focus on prefrontal cortex (PFC) [10–12].

In the present study, we used fNIRS to objectively evaluate malocclusion. We used fNIRS to assess brain activity in the PFC during clenching in the presence of mandibular retrusive deviation, which is one form of malocclusion. The working hypothesis states that (1) the mandibular deviation affects the human emotional state and that (2) using fNIRS, we could evaluate the mandibular deviation objectively focusing on PFC activities.

## 2. Materials and Method 

### 2.1. Subjects

Twelve healthy, right-handed volunteers (seven males and five females, mean age = 29.4 years, range 2.8 years) participated in this study. No subject had a history of neurological or psychiatric illness. All subjects had full dentition and healthy periodontal tissue. Written informed consent was obtained from each subject. The study was approved by the Ethics Committee of Kanagawa Dental University (approval number 251) and conformed to the STROBE guidelines for reporting observational studies (http://www.strobe-statement.org/).

### 2.2. fNIRS

In the present study, fNIRS (ETG7100, Hitachi Medical. Co., Kashiwa, Japan) was used to detect changes in cerebral blood flow. The probe had eight illuminators and seven detectors in a lattice pattern, resulting in 22 channels. The system used two different wavelengths (695 ± 20 nm and 830 ± 20 nm) and measured the level of oxyhemoglobin (oxy-Hb) and deoxyhemoglobin (deoxy-Hb) as well as their sum (total hemoglobin; total-Hb) at a time resolution of 0.1 s. Interoptode distance was 30 mm and the system was able to detect cerebral blood flow at a depth of approximately 20 mm from the scalp [13]. In this study, we used the change in oxy-Hb concentration as an indicator of the change in regional cerebral blood volume. Using a rat brain model, Hoshi et al. showed that that oxy-Hb is more sensitive to brain activity than deoxy-Hb [14].

**Figure 1:**
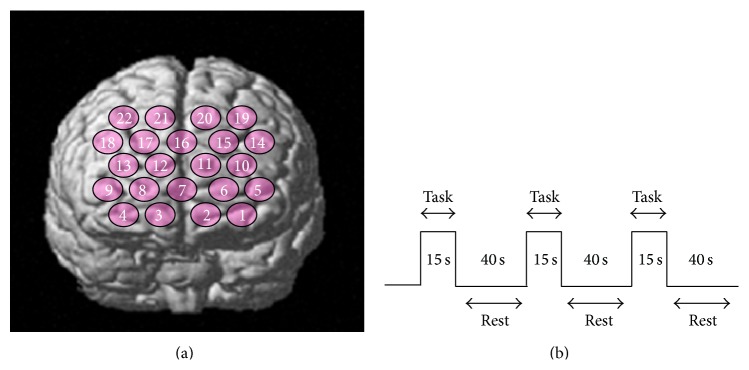
(a) The fNIRS probes were standardized to the frontal region that covered the prefrontal cortex. (b) Schematic illustration of block design.

The fNIRS probes were arranged according to the international 10/20 system used in electroencephalography. The fNIRS probes were standardized to the frontal region that covered the PFC. The lowest probe line was positioned along the Fp1-Fp2 line and the center probe was located at Fpz (Figure 1(a)).

To estimate the correspondence between channels and cortical topography, we employed a probabilistic estimation method [15], which registers fNIRS data to the Montreal Neurological Institute (MNI) standard brain space. fNIRS optode positions and several scalp landmarks were digitized using the 3D magnetic space digitizer (PATRIOT; Polhemus, Colchester, VT, USA). Using an anatomical database [16], channel positions were then estimated in the standardized stereotaxic MNI 3D brain atlas (freeware available at http://www.jichi.ac.jp/brainlab/indexE.html). Based on the anatomical estimation, we selected channels that were located on anterior-dorsal region of the medial PFC (adMPFC). NIRS-SPM software (Bio Imaging & Signal Processing Lab, Daejeon, Korea; freeware available at http://bisp.kaist.ac.kr/NIRS-SPM.html) was also used. fNIRS data (oxy-Hb) were statistically analyzed based on a general linear model and *P* values were calculated. Areas activated in response to the clenching task were identified at uncorrected *P* < 0.05 [17].

### 2.3. Clenching Task

We made mandibular deviation model splints using the same procedure as described in our previous study [1]. Two maxillary splints were produced for each subject, starting with two 0.5 mm thick polyvinyl chloride sheets that were formed to the shape of the maxillary dentition. One of these formed sheets was the control splint. Self-curing dental resin was added to the anterior region of the other formed sheet, and while the resin was still malleable the sheet was fitted onto the subject's maxillary dentition using the chin-point technique, whereby the thumb and one finger were placed on the chin and used to guide the mandible backward and upward so that the mandibular anterior dentition pressed into the soft resin. The resin was allowed to harden, and this unit served as the maxillary splint that would force the subject's condyle into the rearmost position in the mandibular fossa during clenching. The mandibular shift produced by each splint was determined using the SAM condylar position measurement system (Great Lakes Orthodontics, Ltd., Tonawanda, NY). To reduce measurement error, a single experienced technician tested each subject three times.

Each subject performed a maximum voluntary clenching (MVC) task with each splint (retrusion-forcing splint and control splint). With one of the splints in place, the subject performed a series of three cycles of clenching, each cycle consisting of 15 s MVC and 40 s rest (Figure 1(b)). A recovery and reset period of 20–30 min separated the two conditions (retrusion-forcing splint and control splint) for each subject to eliminate the influence of one task on the other.

### 2.4. Data Analysis

Cortical activation was evaluated using the task-related increase in oxy-Hb level. The oxy-Hb data were averaged across the three cycles of the MVC task. NIRS-SPM software (Bio Imaging & Signal Processing Lab) [17] was used to identify the task-related area. To remove the physiological noise, temporal smoothing using Gaussian Kernel and discrete cosine transform (DCT) were used. fNIRS data (oxy-Hb) were statistically analyzed based on a general linear model and *P* values were calculated. Areas activated in response to the clenching task were identified at uncorrected *P* < 0.05. This method identified the anterior-dorsal region of the medial PFC (adMPFC) as task-related area. Probabilistic estimation [15] was performed to confirm the brain area. Based on the anatomical estimation, we selected channels 7, 11, 12, and 16 located on the anterior-dorsal region of the medial PFC (adMPFC), which are our ROI in this study. The mean oxy-Hb level during each condition was calculated. Then, the average across channels 7, 11, 12, and 16 was calculated and compared across conditions using a paired *t*-test.

### 2.5. Visual Analog Scale (VAS)

Each subject rated their subjective feeling of discomfort during the MVC task on a VAS ranging from 0 (no discomfort) to 10 (extreme discomfort). The VAS score was verbally expressed to an interviewer. A paired *t*-test was used to compare the VAS score across conditions, and statistical significance was set at *P* < 0.05. Spearman rank-correlation coefficient was calculated to evaluate the correlation between PFC activation and VAS score for the 12 subjects.

## 3. Results

**Figure 2:**
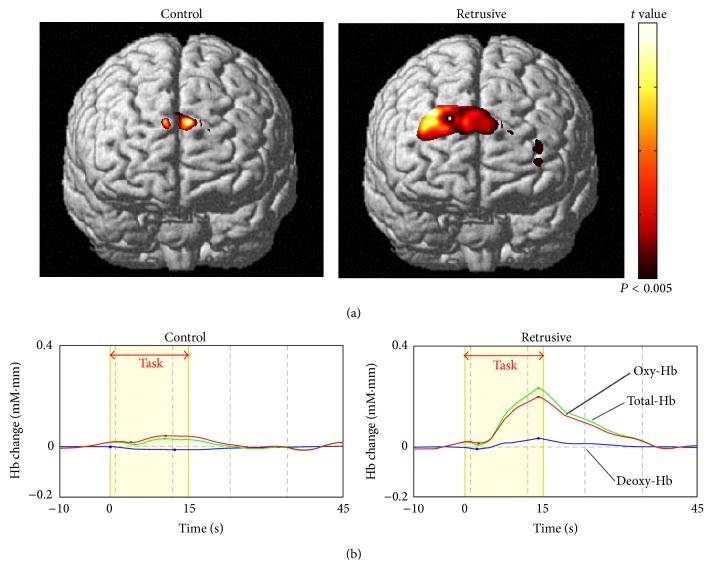
(a) Activation of the prefrontal cortex. Brain activation maps found by group analysis for each clenching task (*n* = 12 participants; *P* < 0.005, uncorrected). Color code denotes *t* value. (b) Time course of the change in hemoglobin in the prefrontal cortex of a representative participant. Task-related change in oxy-Hb (red), deoxy-Hb (blue), and total-Hb (green). Red arrow represents the clenching task.

Figure 2(a) shows the results of the NIRS-SPM spatial analysis for a typical subject. The adMPFC was activated during both control and retrusive position clenching. The activated area was larger in retrusive position clenching than in control position clenching. Probabilistic estimation [15] indicated that channels 7, 11, 12, and 16 were located over Brodmann area 10, which corresponds to the anterior-dorsal region of the medial PFC (adMPFC; Table 1).

Figure 2(b) shows the time course of the change in oxy-Hb, deoxy-Hb, and total-Hb level. There was an increase in oxy-Hb and total-Hb in the PFC during both control and retrusive position clenching. The increase in oxy-Hb was greater in retrusive position clenching than in control position clenching. After clenching stopped, oxy-Hb gradually returned to baseline.

**Figure 3:**
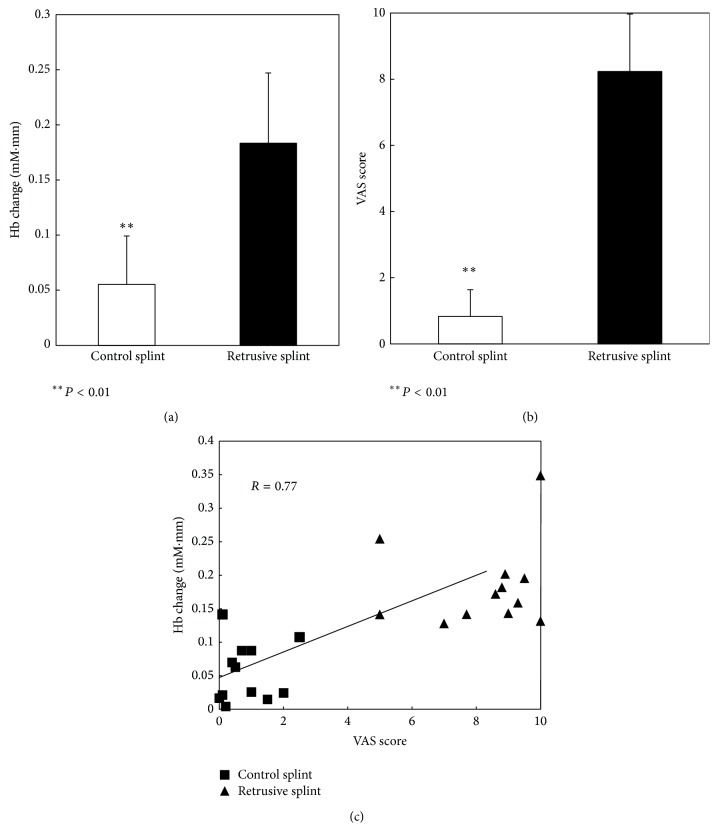
(a) Change in Oxy-Hb in the prefrontal cortex during the clenching task performed with the control splint and the retrusive splint. (b) Mean VAS score for discomfort. Error bars indicate SE. (c) The relation between the change in oxy-Hb in the prefrontal cortex and the VAS score for discomfort in the clenching task performed with the control splint and the retrusive splint.

Figure 3(a) shows the change in oxy-Hb concentration in the adMPFC in control and retrusive position clenching. The comparison between control and retrusive position clenching revealed significantly greater activation during retrusive position clenching (*P* < 0.01).  Figure 3(b) shows the VAS score for discomfort in control and retrusive position clenching. The comparison between control and retrusive position clenching revealed significantly larger VAS score for discomfort during retrusive position clenching (*P* < 0.01).  Figure 3(c) shows the correlation between the change in oxy-Hb and the VAS score for discomfort. There was a statistically significant positive correlation between the change in oxy-Hb and the VAS score for discomfort (*R* = 0.77).

## 4. Discussion

To the best of our knowledge, this is the first study to investigate the relation between malocclusion and brain activation using fNIRS. Previous fMRI studies have suggested that PFC activation correlates with the severity of malocclusion [2]. In the present study, clenching with the malocclusion model (the mandibular retrusive condition) caused PFC activation. Hoshi et al. reported that pleasant or unpleasant emotions could be recognized from cerebral blood flow evaluated using fNIRS [11]. The PFC is closely associated with emotional stress and negative emotional reactions [10–12, 18] and is involved in various high-level cognitive functions [19–22]. Yasui et al. reported that oxy-Hb concentration, particularly in the adMPFC, reflected the level of mental stress and the activity of the autonomic nervous system [10]. In this study, there was a statistically significant positive correlation between prefrontal blood flow and VAS score for discomfort. This indicates that PFC activity in the present study probably played a role in the rating of unpleasantness.

In this study, we used fNIRS to observe the brain activation during clenching. fNIRS is affected by muscle activation. Therefore, the present results may be affected by temporal muscle activation. Narita et al. used principle component analysis to investigate PFC activation during gum chewing [23]. Principle component analysis enables the effects of muscle activation to be eliminated from the fNIRS signal. In this study, we did not use principle component analysis, and our results may therefore be affected by temporal muscle activation. However, in this study, we observed a tendency for an increase in oxy-Hb and a decrease in deoxy-Hb during the retrusive position condition. These results are consistent with previous fNIRS studies that showed an increase in oxy-Hb and a decrease in deoxy-Hb during brain activity [8, 13]. Therefore, we assume that the changes in hemoglobin observed using fNIRS in this study reflect brain activation.

Our previous studies suggested that fMRI was a useful diagnostic device for functional occlusion [1, 2]. However, an fMRI device requires a large, shielded room and has a high cost, so it is difficult to routinely use fMRI in the dental clinic. By contrast, the fNIRS device is relatively inexpensive compared to other neuroimaging methods. The spatial resolution is typically coarse, at 3 cm or more, but may improve with technical advancements, and the temporal resolution can be quite high. fNIRS has the particular advantage of being able to monitor a moving patient, which would be particularly suited to dental practice. The clinical significance of this study is applicable to dental practice the neuroimaging tool such as fNIRS.

## 5. Conclusion

The results of the present study suggest that fNIRS of the PFC could be used to objectively evaluate the occlusal condition. Because the fNIRS device is easier to control than other neuroimaging methods, it is more suitable for evaluating the occlusal condition in clinical settings. However, the exact link between malocclusion other than mandibular retrusion and activation of the PFC is unclear, because malocclusions vary widely and the sample size is not large enough to permit a definitive conclusion at the present time. Further research is required.

## Figures and Tables

**Table 1:** Channel location.

Channel	MNI coordinates (mm)			SD (mm)	Brodmann area
	*X*	*Y*	*Z*		
7	−8	73	11	9.5	10
11	−17	69	21	7.0	10
12	9	70	22	9.0	10
16	−7	64	33	8.0	9, 10
